# Nano-Porous-Silicon Powder as an Environmental Friend

**DOI:** 10.3390/ma14154252

**Published:** 2021-07-30

**Authors:** Marwa Nabil, Kamal Reyad Mahmoud, Raghda Nomier, El-Maghraby El-Maghraby, Hussien Motaweh

**Affiliations:** 1 Department of Electronic Materials Researches, Advanced Technology and New Materials Research Institute, City for Scientific, Research and Technology Applications, New Borg El-Arab City 21934, Egypt; 2 Department of Physics, Faculty of Science, Kafrelsheikh University, Kafr El Sheikh 33516, Egypt; kamalreyad@gmail.com; 3 Department of Physics, Faculty of Science, Damanhour University, Damanhur 22511, Egypt; raghda.mhmd91@yahoo.com (R.N.); maghrabym@yahoo.com (E.-M.E.-M.); prof_motaweh@yahoo.com (H.M.)

**Keywords:** microporous materials, positron annihilation spectroscopy, X-ray diffraction

## Abstract

Nano-porous silicon (NPS) powder synthesis is performed by means of a combination of the ultra-sonication technique and the alkali chemical etching process, starting with a commercial silicon powder. Various characterization techniques {X-ray powder diffraction, transmission electron microscopy, Fourier Transform Infrared spectrum, and positron annihilation lifetime spectroscopy} are used for the description of the product’s properties. The NPS product is a new environmentally friendly material used as an adsorbent agent for the acidic azo-dye, Congo red dye. The structural and free volume changes in NPS powder are probed using positron annihilation lifetime (PALS) and positron annihilation Doppler broadening (PADB) techniques. In addition, the mean free volume (VF), as well as fractional free volume (Fv), are also studied via the PALS results. Additionally, the PADB provides a clear relationship between the core and valence electrons changes, and, in addition, the number of defect types present in the synthesized samples. The most effective parameter that affects the dye removal process is the contact time value; the best time for dye removal is 5 min. Additionally, the best value of the CR adsorption capacity by NPS powder is 2665.3 mg/g at 100 mg/L as the initial CR concentration, with an adsorption time of 30 min, without no impact from temperature and pH. So, 5 min is the enough time for the elimination of 82.12% of the 30 mg/L initial concentration of CR. This study expresses the new discovery of a cheap and safe material, in addition to being environmentally friendly, without resorting to any chemical additives or heat treatments.

## 1. Introduction

The major threat, for the time being, which must be dealt with on a global level is toxic and carcinogenic environmental pollutants. In particular, the new technologies developed for the easier decolorization of different compound types have attracted widespread interest [1]. Many industries produce residual dyes (i.e., dye intermediates, textile, paper, and pharmaceutical industries, etc.). Wastewater treatment systems have to deal with a wide range of organic pollutants. Pollution with dyes is undesirable, as many of the dyes released are toxic and carcinogenic [2]. In order to remove the wastewater color, several physical and chemical experiments have been performed. Therefore, it was found that the process of de-pigmentation using physical adsorption technology is the most effective and economically appropriate [3]. So, the adsorption technique is one of the best techniques for water reuse, as a result of its economic cost, simple design, ease of operation and non-toxicity [4]. Accordingly, many porous adsorbent materials, such as activated carbon [5], peat, chitin, and silica, are used for testing the possibility of dye removal [6]. However, intraparticle diffusion associated with porous adsorbents may lessen the rate and capacity of adsorption [7]. Therefore, the adsorption process is a surface process; its adsorption value and its specific surface area are directly proportional to each other [8]. The ratio between the rising surface area and nano-adsorbent mass of materials can promote the sorbent material’s adsorption capacity.

Generally, NPS material is a network containing a homogenous mixture of air and silicon. From the optical point of view, NPS is specified as an effective medium, and it is considered environmentally suitable for use as an adsorbent material. Its optical properties rely on the silicon prorated volumes, and the pore filling medium [9]. The ultra-sonication technique is one of the most famous materials processing techniques that is widely used for powder technology, as a result of its simplicity and effectiveness, as shown in previous research [10].

The PALS is an important tool and non-destructive technique that has been used for the characterization and investigation of the microstructural properties of different materials. Positron experiments confirmed the sensitivity of PALS to the studied defects in metals/alloys, and free volume/pores in molecular solids [11]. Additionally, in the case of porous materials, the formation of positrons are implanted from a radioactive source in the molecular solids, and each pore of them annihilates with e^-^ of the material’s atoms and for the formation of a positronium (Ps), s shown in previous studies. Thus, the pluck annihilation rate (also known as the lifetime) is correlated with the pore size in the simple free-volume model size according to the simple free-volume model [12,13,14,15,16].

Doppler broadening spectroscopy (PADB) supplies valuable information regarding the inner electronic shells’ contribution and provides valuable data about chemical annihilation. The S-parameter is defined as the ratio of counts in the central part of the Doppler broadened spectrum to the area below the annihilation line completely. It depends on the average density of volume defects, which is open. On the other hand, the ratio between the area below the annihilation line fixed-wing region and the area under the whole annihilation line is defined as W [17]. This is related to the positron annihilation with deeply bound core electrons, which provides information about the chemical environment of the defect. Thus, the PALS technique obtains the e^-^ density data at the positron annihilation site, and the PADB methods provide information on the momentum distribution of electrons. All of them are widely used in modern materials science. Several studies, in particular on solids and porous systems, have included NPS and nonporous SiO_2_ via the PALS technique [18,19,20,21,22,23,24,25].

As shown in our previous research [26,27,28,29,30], the solid’s nano-scale microstructure is studied. We report herein the application of the PALS for tracking the free volume size changes for synthesized NPS powder via a combination of the alkali chemical etching process and the ultra-sonication technique, starting with commercial silicon powder. Additionally, this work is targeted at studying acidic Congo red dye removal from aqueous solutions using synthesized NPS powder via the adsorption process.

## 2. Materials and Methods

### 2.1. NPS Powder Production and Characterization

The combination of two techniques (ultra-sonication and alkali etching process) for NPS powder production was performed, starting with commercially available Si-powder (Silicium, Pulver—99%, Burlington, VT, USA), as shown in previous studies. A suitable amount of Si powder was dispersed in n-propanol and KOH was dispersed in distal H_2_O. The product powders were filtrated, washed, and then dried overnight.

The construction and crystallization of the synthesized NPS were analyzed using XRD (X-ray 7000 Shimadzu diffractometer, Kyoto, Japan). (Fourier transform infrared spectrophotometry (FTIR-Shimadzu FTIR-8400 s) was used to determine the NPS powder forming chemical bonds. In addition, high-resolution transmission electron microscopy (HR-TEM, Tecnai G20, FEI, Eindhoven, The Netherlands) was utilized in the description process of the NPS powder product’s morphology.

### 2.2. Positron Annihilation Lifetime Measurements

This work used the spectrometer due to its fast spectrometry [31], with a resolution of ~350 ps via the ^60^Co source at room temperature for measurements of the lifetime of the positron. To study the activity of 15 µCi of ^22^Na, the sample was deposited and dehydrated upon Kapton foil (7.6 µm thick), and then glued using epoxy glue. During the measurements, this assembly was sandwiched between two similar samples as a positron source. The measurement of each sample was repeated at least 2–3 times, and the total number of elementary annihilation events was approximately 1–2 million. The LT computer program from Kansy was used to resolve the collected spectra. [32]

As a result of measured spectra analysis, there are 3 lifetime items (τ1, τ2, and τ3). The 1st lifetime item τ_1_ is produced due to the P-positronium (p-Ps) atom (fixed at 0.125 ns). The 2nd lifetime item τ_2_ is produced during the positron annihilation via free electrons inside the material. Finally, the 3rd lifetime item (τ_3_), which is the longest lifetime component, depends on the ortho-positronium (o-Ps) annihilation via the “pick-off” mechanism in the amorphous regions free volume sites. All items were determined via the fit’s variance (1.005 to 1.18). So, τ_3_ provides valuable data regarding the free volume cavities’ mean size when probed by o-Ps.

For the free-volume model [12], the o-Ps lifetime focused inside a spherical solid potential well (radius = R_o_) and the free volume of radius R, and no electrons were found below it, as shown in the following equation [33,34]:

τ_o_ − P_s_ = 0.5 × [1 − (R/R_O_) + (1/2π) Sin (2πR/R_o_)]^−1^ (1)

where δR = R_o_−R = 1.656 Å is the fitted empirical electron layer thickness. With this value of δR, the free volume radius (R) was calculated from Equation (1), and the average size of the free volume holes (V_f_) was calculated as V_f_ = (4/3) πR_3_ (in Å^3^).

Furthermore, the free volume hole fraction, f_v_, can be estimated using the empirical equation [35]:

f_y_ = CV_f_I_3_ (2)

where V_f_ is in angstrom cube, I_3_ in percent, and C is an arbitrarily chosen scaling factor for a spherical cavity.

### 2.3. Doppler Broadening Measurements

Using a Ge-detector (Ortec, p-type high-purity, GEM series, Oak Ridge, TN, USA), Doppler broadening was measured. Its energy resolution was FWHM = 1.6 keV for the γ-line (1.33 MeV) of ^60^Co. A relative efficiency of 25% was applied for defining S and W as the line-shape parameters of the Doppler broadening. Ortec 570 was used for magnifying the detector output signals, which were then obtained via an Ortec 919 multichannel analyzer (MCA). A 5 µCi ^22^Na sample was prepared via a droplet of NaCl solution that was dried on 2 congruent Kapton foils, and then glued using epoxy glue. Both samples (disks) were coordinated with the ^22^Na source in a 4π configuration. The energy was calibrated (~68 eV/channel) via the ^133^Ba source. The Doppler broadening spectra were taken until more than two million counts had accumulated in the peak. These measurements were performed in air at room temperature. The obtained Doppler broadening spectra were analyzed using the SP ver. 1.0 program. The calculation of the S- and W-parameters, depending on the centroid channel with maximum counts of the 511 keV peak, was accurately determined. The input data were fixed for all spectra of the studied samples.

### 2.4. Dye Decolorization Using the Batch Procedure

The wastewater was synthesized by dissolving the acidic Congo red dye in distilled water to gain the required waste solution concentrations; this was applied to monitor the efficiency of the NPS product’s adsorption. Then, 30 mL of wastewater solution (10 and 50 ppm) was mixed with 0.15, 0.3, 0.6 and 1 g of the NPS product for 15 min using the orbital shaker. The solid phase was separated from the solvent phase using a centrifugation technique (600× *g* rpm for 15 min). The remaining acidic Congo red concentration was analyzed using a UV-Visible spectrophotometer at a wavelength of 486 nm.

The residual mass comprised adsorbed metal ions; the collected filtrate was exposed to metal ion assessment via the UV-Visible Spectrophotometer Double Auto Cell (Labomend. INC, Los Angeles, CA, USA). We then calculated the percentage of metal uptake, using the sorption efficiency, and then the amount of metal ions that were adsorbed [36]. The tests were executed to determine the impact of contact time (20–60 min) and the temperature of the waste solution (25 °C).

Sorption efficiency = (C_i_ − C_f_)/C_i_ × 100 (3)

Amount Adsorbed (Q_e_) = (C_i_ − C_f_)/W × V (4)

where C_i_ is the initial metal ion concentration in the solution (mg/L), C_f_ is the final metal ion concentration in the solution (mg/L), W is the adsorbent weight (g), V is the solution volume (L), and Q_e_ is the amount of metal ions that adsorbed per gram of adsorbent.

## 3. Results and Discussion

### 3.1. NPS Powder Characterization

The chemically prepared NPS powder material was examined via various physicochemical techniques in order to investigate its structure and properties.

#### 3.1.1. X-ray Diffraction Analyses

**Figure 1:**
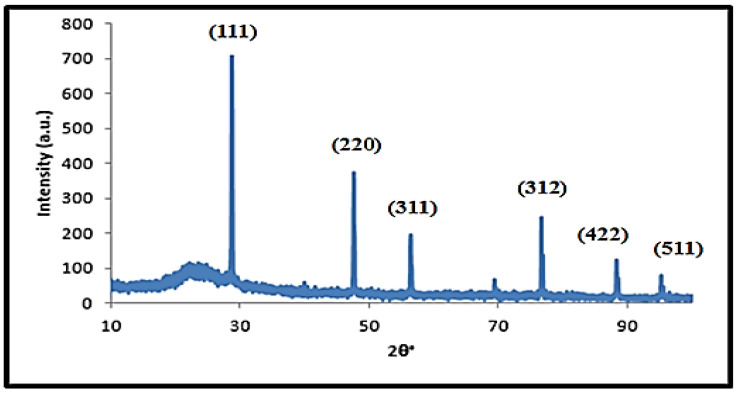
X-ray diffraction pattern of the prepared NPS powder.

Figure 1 describes the diffraction peaks of the NPS powder product which is perfectly reported in the cubic phase NPS (JCPDS Card No. 01-079-0613 and 00-027-1402). The strongest peak appears at 2θ = 28.23°, which corresponds to (111), while other peaks appeared at 2θ = 47.193°, 56.023°, 76.261°, 87.9382° and 94.8370°, which correspond to (220), (311), (312), (422) and (511), respectively. It is also noted that limited silica formation occurs at 2θ = 23.128°. There are no impurity peaks in the pattern, meaning that it is corroborative of the high purity of the prepared NPS powder.

#### 3.1.2. Fourier Transform Infrared Spectroscopy (FTIR)

**Figure 2:**
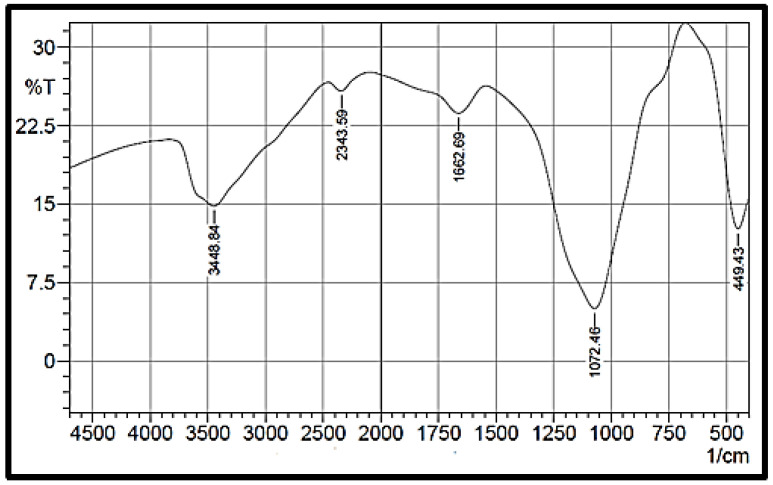
FTIR spectrum of the prepared NPS powder.

Figure 2 presents the NPS product’s FTIR spectrum in the range 400–4000 cm^−1^. The peaks within the wavelength range of 1000–1300 cm^−1^ are assigned to Si–O asymmetric stretching in Si–O–Si, and the peak at 449 cm^−1^ corresponds to Si–O bending. In addition, the formation of an NPS product peak is recorded at 1072 cm^−1^. The broad peak at 3449 cm^−1^ corresponds to the presence of interstitial water and the hydroxyl group. The peak at 1662 cm^−1^ corresponds to the free water molecules’ deformation vibration [37]. Therefore, the FTIR spectrum agrees that the product is pure NPS with no pollutants due to oxidizing and wetting agents, which were utilized in the preparation step.

#### 3.1.3. Transmission Electron Microscopic Analyses (TEM)

**Figure 3:**
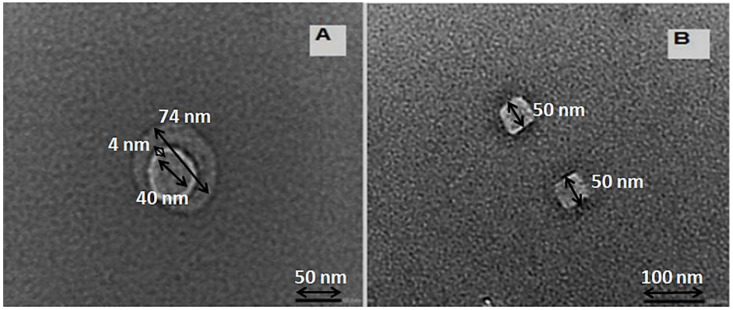
TEM images for NPS and nano porous silica powders in the preparation conditions—7 g of commercial Si powder, 3 wt.% KOH, and at sonication times; (**A**) 3 h, and (**B**) 4 h.

TEM images of the prepared NPS powder are shown in Figure 3. In the preparation conditions—7 g of commercial Si powder, 3 wt.% KOH at sonication times of 3 and 4 h—the morphological construction of the NPS powder product as illustrated in Figure 3A has a spherical NPS morphology covered with a nano-silica layer. In the case of Figure 3B, the TEM image presents the cubic shape with good crystallinity. Figure 3 shows that the NPS product is in the nano range. These results provide a prediction of the NPS powder product, which has a huge surface area, which is useful for enhancing its dye pollutant removal affinity.

### 3.2. Positron Annihilation Lifetime (PAL) Parameters

The spectra of positron lifetime are classified in terms of three items of positron lifetime, τ_1_, τ_2_, τ_3_, while the intensities are I_1_, I_2_, I_3_ for the NPS powder product, respectively. Due to the poor resolution time of the apparatus (≈350 ps), the short-lived item data for the p-Ps are unreliable. For the minimization of the scatter of the other parameters, τ_1_ was fixed at 125 ps. Accordingly, the suitable quality of the spectra did not change and the derived parameters were close to those obtained when the analysis was made without any restrictions.

For all measured samples, the intermediate-lifetime component (τ_2_ = 0.351–0.497 ns) and its relative intensities (I_2_) ranged from 29.00% to 44.2%, as shown in Table 1. Additionally, it may arise from the interaction of positrons with e^-^s placed in higher negative charge density. The τ_2_ and I_2_ values are found to be in the same order as those commonly seen in the literature [20,24]. The longest-lived one, τ_3_, may be attributed to the o-Ps annihilation localized in nano-regions, within the silica matrix, which is very sensitive to the microstructural changes. In molecular systems, the o-Ps localized in a cavity annihilate via a pickoff annihilation technique with an antiparallel electron spin from the cavity wall surroundings. The τ_3_ determination provides valuable information on the mean size of free volume cavities probed by o-Ps.

Table 1 contains the calculated values of the τ_3_ and its relative intensity I_3_ that classifies the annihilation parameters of the o-Ps as a function of sonication time (2, 3, and 4 h) at the preparation conditions (commercial silicon weight (5 and 7 g) and several KOH_conc._ (3, 4.5 and 6 wt.%)). Additionally, samples at a sonication time of 4 h and special conditions without heat treatment (No. H) or without filtration (No. F) are presented. The range of the longest-lived item, τ_3_, is 1.31–2.19 ns, and its corresponding intensity (I_3_) in within the range 1.63–21.5%, for all the measured samples. The values of t_3_ and I_3_ are the smallest [20,21,38,39].

It is clear from Table 1 that there is a directly proportional relationship between the sonication time and the values of I_3_%). This is a logical relationship, which is a result of the enhancement of the porosity percentage in Si powder for NPS formation. One can notice that a surprising enhancement of the I_3_% values was shown in samples with 4 h sonication time with special conditions of no heat treatment (No. H) or no filtration (No. F). In these processes, a longer period time of the oxidized agent was achieved as a result of the NPS surface oxidation process. Consequently, the porous silica layer was formed on the NPS core, which has a larger surface area, increasing the porosity percentage and decreasing the o-Ps lifetime (τ_3_ ns), and consequently decreasing the size of the free volume, as shown in Table 1. This result was proven and in good agreement with the TEM measurements (see Section 3.1.3).

**Figure 4:**
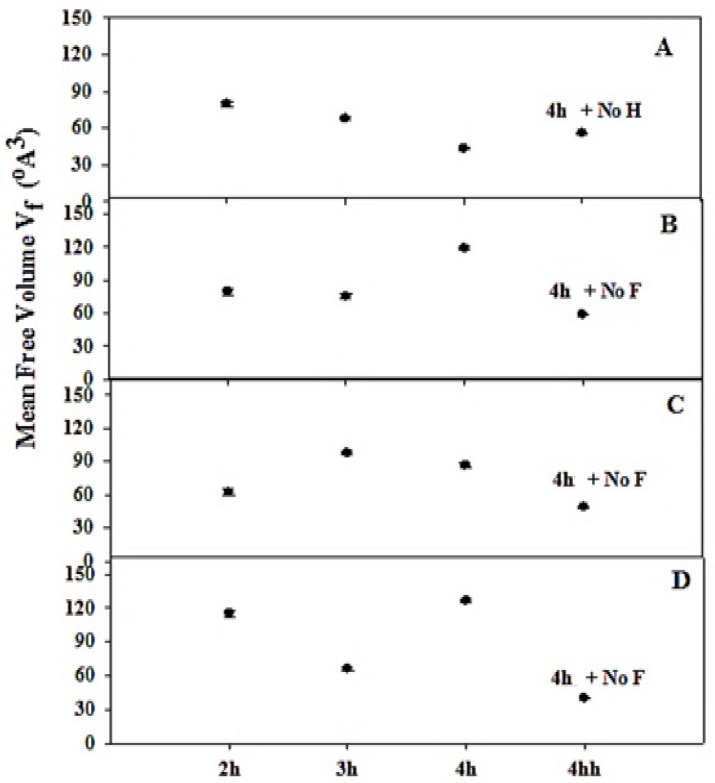
The variation of mean free volume, Vf (Å3), as a function of sonication time (2, 3 and 4 h) in special conditions; 7 g commercial silicon powder in different KOH concentrations (wt.%): (**A**) at 6 wt.% KOH, (**B**) 4.5 wt.% KOH, (**C**) 3 wt.% KOH and (**D**) 5 g commercial silicon powder and 3 wt.% KOH.

The calculated values of the o-Ps lifetime (τ_3_ ns) in Table 1 were used to calculate the radius R of the free volume V_f_ (Å^3^) according to the free-volume model [12]. Figure 4 shows the variations of mean free volume V_f_ (Å^3^) as a function of sonication time (2, 3, and 4 h) for the measured samples at weight of commercial silicon powder (5 and 7 g) in different KOH concentrations (6, 4.5, and 3 wt.%). In addition to samples at a sonication time of 4 h in special conditions and with a slow drying process (without heat treatment (4 h + No. H)) and without the separation process (without filtration (4 h + No. F)). It is clear from Figure 4 that the effect of sonication time on the mean free volume V_f_ (Å^3^) has the same trend as the o-Ps lifetime, τ_3_ (ns) (as shown in Table 1), and also the same explanation can be suggested. It can be concluded that a severe reduction in mean free volume was observed in samples at a sonication time of 4 h in the special conditions and with a slow drying process, without filtration as a result of the formation of a silica layer on the NPS material.

**Figure 5:**
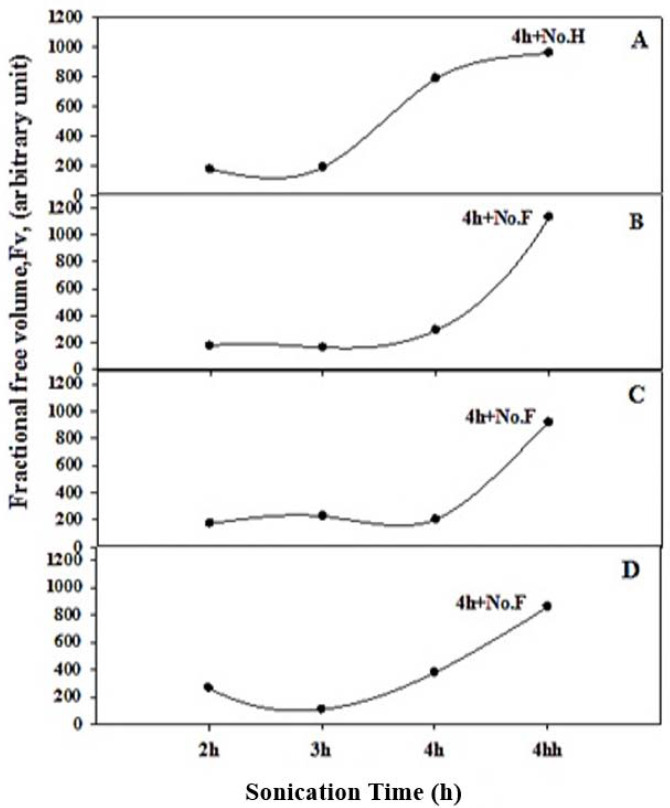
The variation of fractional free volume, Fv, as a function of sonication time (2, 3 and 4 h) in special conditions; 7 g commercial silicon powder in different KOH concentrations (wt.%): (**A**) at 6 wt.% KOH, (**B**) 4.5 wt.% KOH and (**C**) 3 wt.% KOH and (**D**) 5 g commercial silicon powder and 3 wt.% KOH.

The variation of the fractional free volume (F_v_) of the NPS samples as a function of sonication time (2, 3 and 4 h) for all the measured samples are also shown in Figure 5. The results show a small increase in the values of F_v_ with a sonication time of 2, 3 and 4 h for all different KOH concentrations, then a steep increase at a sonication time of 4 h in the special conditions and with a slow drying process (without heat treatment (4 h + No. H)) and without the separation process (without filtration (4 h + No. F)). This enhancement may be attributed to the formation of a silica layer on the NPS material.

### 3.3. Doppler Broadening Spectroscopy Measurements

**Figure 6:**
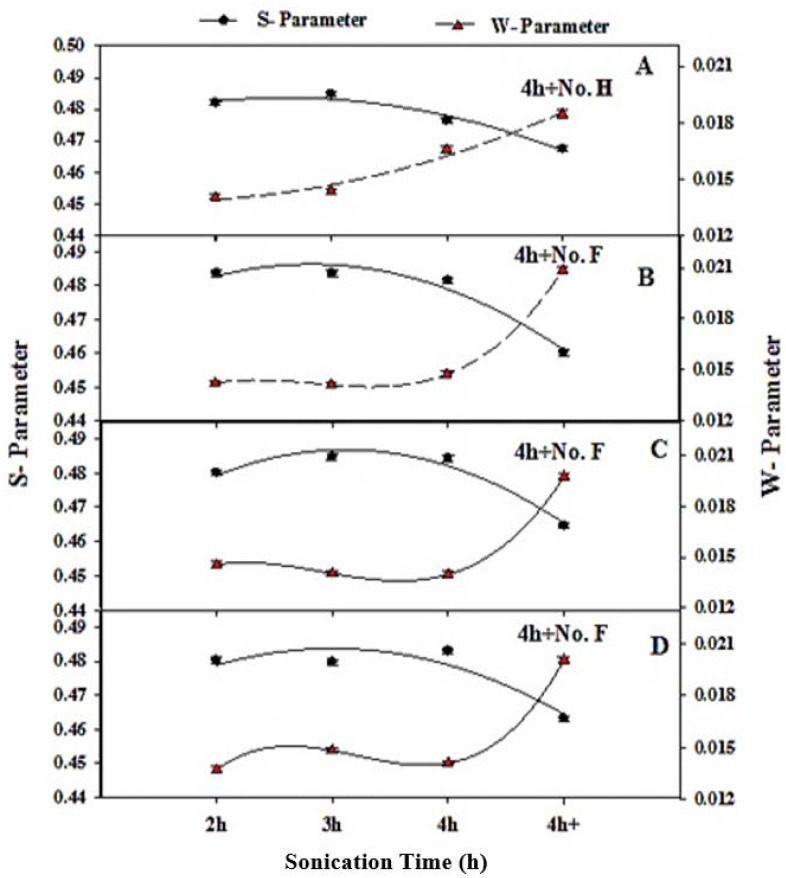
The variation of W–parameters at 7 g commercial silicon powder at sonication time (2, 3 and 4 h) with special conditions with different weights of KOH (wt.%): (**A**) at 6 wt.% KOH, (**B**) 4.5 wt.% KOH and (**C**) 3 wt.% KOH and (**D**) 5 g commercial silicon powder and 3 wt.% KOH.

Although the PALS results are strongly indicative of a long-lifetime component task Ps, the results show this conclusion via another distinct technique, such as Doppler broadening of annihilation radiation (DBAR). The sharpness of an annihilation peak of 511 keV can be measured by the so-called S-parameter, which is an indicator of the fraction of positrons annihilating with the valence electrons. This can be produced when positrons annihilate in vacancies or pores and/or o-Ps annihilate in free volumes with low-kinetic momentum electrons of the outer orbital of the neighboring atoms present at the wall of the pores or the free volumes inside the materials. The estimated S- and W-parameters’ values as a function of sonication time are shown in Figure 6.

The results show that the S-parameter values decreased. However, there is a directly proportional relationship between the W-parameter and sonication time at 7 g commercial silicon powder with 6 wt.% of KOH, as shown in Figure 6A. On the other hand, there are a few variations of the S– and W–parameters for the samples of 7 g commercial silicon powder with 4.5 and 3 wt.% of KOH and 5 g of commercial silicon powder with 3 wt.% of KOH at the sonication times 2, 3, and 4 h, as shown in Figure 6B–D. These variances are in agreement with the PALS information, due to the existence of the Ps; the distribution of momentum is correlated with Ps (o-Ps and p-Ps). This is thinner than that linked with the e^+^ annihilation (“free” positron gain). Thence, the overall line width has to increase at the Ps intensity decreases, and vice versa, as is actually observed.

**Figure 7:**
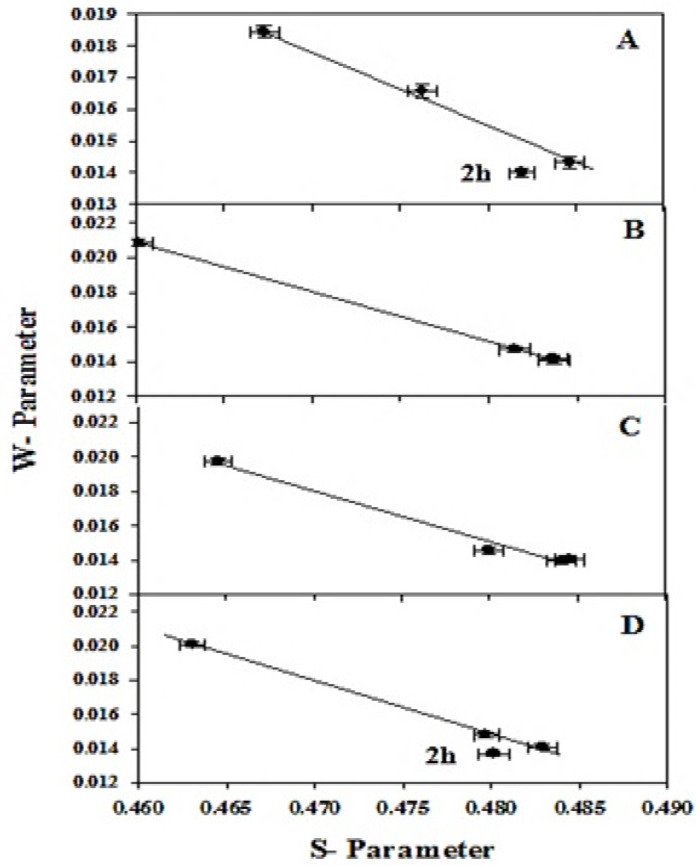
The S versus W plot for 7 g commercial silicon powder at sonication times of 2, 3 and 4 h with special conditions in different weights of KOH (wt.%): (**A**) at 6 wt.% KOH, (**B**) 4.5 wt.% KOH and (**C**) 3 wt.% KOH and (**D**) 5 g commercial silicon powder and 3 wt.% KOH. The solid line represents a linear fitting of the experimental data.

A falling behavior compared with the steep growth of S- and W-parameters, respectively, was recorded for samples in the special conditions of 4 h without filtration, as seen in Figure 6B–D. The steep decrease is perhaps as a result of valance e^-^’s reduction, defect size, and the concentration of particles [31]. Figure 7 shows the defect type number that is obtained by plotting the S-parameter verses the W-parameter. For a sample with one kind of defect, the plot of S against W is linear. From these figures, one notices that the W-parameter is inversely proportional to the S-parameter values for all samples. Thus, only one kind of defect exists in these samples. As shown in Figure 7A,D, the only exception was found in the samples with 7 g commercial silicon powder with 6 wt.% KOH and 5 g commercial silicon powder with 3 wt.% KOH at a sonication time of 2 h.

### 3.4. Basic Dyes Decolourization Process onto the Synthesized NPS Powder Using a Batch Adsorption Technique

#### 3.4.1. Effect of Contact Time

**Figure 8:**
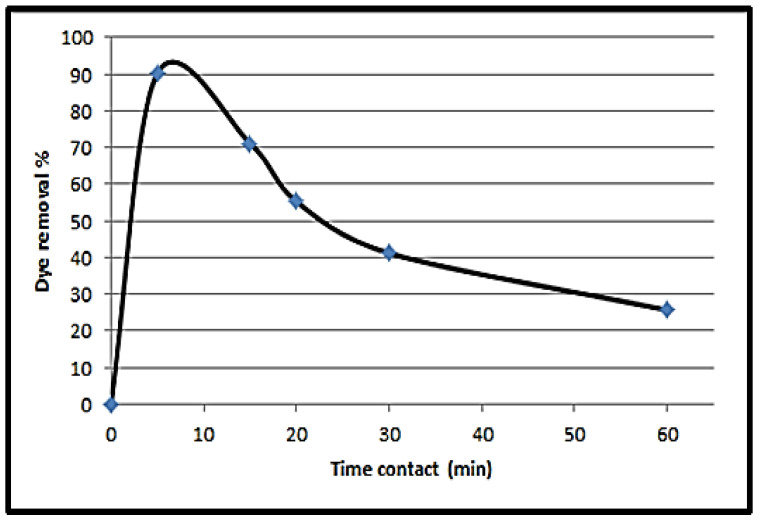
The contact time impact for Congo red removal using NPS (pH = 7; adsorbent dose = 10 g/L; initial CR concentration = 10 mg/L).

The contact time effect on the basic Congo red (CR) adsorption onto the NPS powder surface is presented (Figure 8). The experiments are performed at an initial dye concentration of 10 ppm, with 10 g/L of NPS as an adsorbent, and with a 600 rpm agitation speed at several time interval ranging from 0 to 60 min. It is stated that the CR adsorbed amount is directly proportional to the contact time, and at 5 min reaches its maximum value. The equilibrium time can be considered at 15 min for ensuring the full dye sorption atop the prepared NPS. Therefore, the maximum dye removal above the synthesized NPS powder occurred within 5 min, and subsequently, the system reaches an equilibrium point.

#### 3.4.2. Effect of NPS Powder Dosage

**Figure 9:**
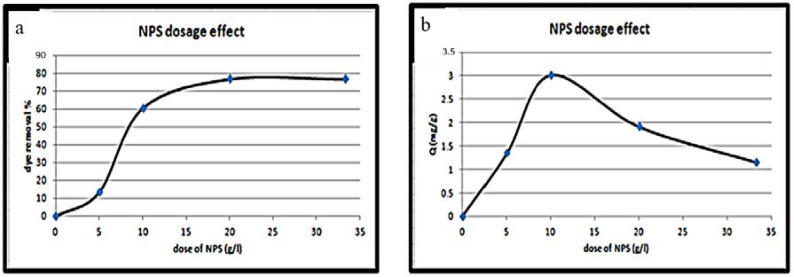
Effect of NPS dosage on: (**a**) CR dye removal, (**b**) the quality of the adsorption process, at initial dye concentration = 50 ppm, temperature = 298 K, pH = 7, contact time = 15 min).

The NPS dosage is an important factor that sets the NPS adsorption capacity at an initial CR concentration of 50 ppm. The CR removal percentage via various NPS dosages and the equilibrium sorption capacity is illustrated in Figure 9a,b. From this figure, the direct proportionality between the NPS dosage value and the removal percentage of CR dye is noticeable. Furthermore, the amount of CR removed per gram of NPS powder tends to reduce with the enhancement of its amount. When raising the NPS dosage at the CR dye concentration of 50 ppm, it supplies a more exposed area for dye adsorption, and thus leads to the enhancement of the extent of CR removal. Otherwise, the amount CR dye removed per gram of NPS reduces; essentially, this is due to the presence of NPS sites, and the rest being unreacted due to the dye’s adsorption. Furthermore, regarding the prepared NPS dosage (over 10 g/L), a trivial increase was recorded with the increase in NPS dosage up to 33.3 g/L. Thus, 10 g/L of the NPS is chosen as the optimum adsorbent material dosage for CR dye removal.

#### 3.4.3. Initial Dye Concentration Impact

**Figure 10:**
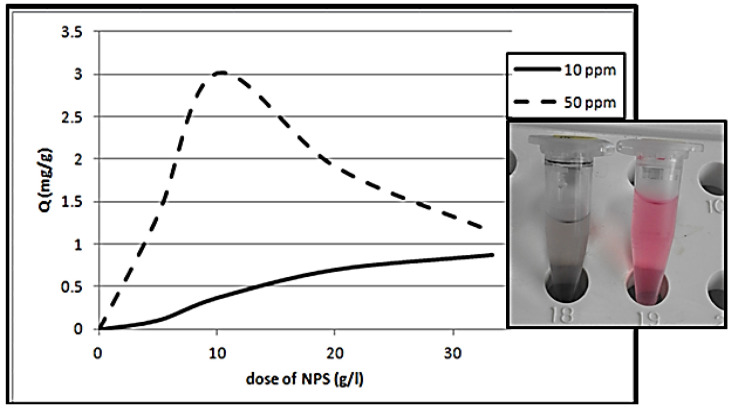
Impact of CR initial concentration on the adsorption values (at different adsorbent doses; T = 298 K; pH = 7; t = 15 min).

The quantitative analysis of the CR removal percentage at equilibrium on the NPS surface at various initial dye concentrations is presented in Figure 10. It clarifies the inversely proportional relationship between the dye adsorption percentages and the initial dye concentration. Moreover, the adsorbed amount of dye per adsorbent unit mass is affected by raising the concentration of the initial dye. 

At high dye concentrations, the dye adsorption onto the prepared NPS reduced. This is as a result of the ratio value; the initial mole number of the dye to the adsorbent material’s surface area. Hence, the fractional factor between the adsorbent and the adsorbate is dependent on the adsorption process. Then, the dye concentration initially supplies a significant driving force to overcome the resistance of the dye mass transfer between both aqueous and solid phases. So, at the highest initial dye concentration, the ion number for the available sites on the NPS surface is high too, ameliorating the basic CR adsorption capacity [40,41,42].

Furthermore, the impacts of the temperature and acidity on the value of CR adsorption are studied. When changing the pH and temperature values, no obvious impact on the CR adsorption values is observed. At room temperature and pH = 7, no change in the removal percentage can be seen compared to the previous cases. Then, all the prepared samples (as previously mentioned in Table 1) are tested in the batch technique for dye adsorption. However, the result was negative, except for the samples which were prepared at the preparation conditions of 7 g commercial silicon powder, with a sonication time of 4 h with a slow drying process (without heat treatment (4 h + No. H)) and without a separation process (without filtration (4 h + No. F)). This agrees with the results of the free volume values (I_3_), which agree with the PALS measurement values. The PALS measurements and the TEM images also agree with the final results of the CR adsorption process.

## 4. Conclusions

The morphological and crystalline description of the NPS product records the high purity state with good crystallinity. The NPS powder is prepared using the combination of two techniques. The PALS results show that positron annihilation can be a useful technique to characterize the NPS product. The results indicate an enhancement of the I_3_% values at a sonication time of 4 h; without heat treatment (No. H) and without a filtration process (No. F). The produced results are in line with the TEM measurements. DBAR measurements show an inversely linear relationship between S and W for all samples. This suggests that only one type of defect is present in these samples.

The NPS product is used effectively for CR dye adsorption from aqueous solutions. The dye removal percentage is reinforced at the increased contact time value. The best dye removal occurs at 5 min, and afterwards the equilibrium point is reached by the system. The best CR adsorption capacity of the NPS product is 2665.3 mg/g, at an initial CR concentration of 100 mg/L and an adsorption time of 30 min, with no pH and temperature effect. Therefore, 5 min is sufficient for removing 82.12% of CR at an initial concentration of 30 mg/L.

## Figures and Tables

**Table 1:** The results of lifetime components with the corresponding intensities at 7 and 5 g of commercial silicon powder in different weight of KOH (wt.%) at sonication times of 2, 3, and 4 h).

Sample Composition	Sonication Time (h)	τ_2_ (ns)	τ_3_ (ns)	I_1_ (%)	I_2_ (%)	I_3_ (%)
**7 g Si Powder + 6 wt.% KOH**	2	0.359 ± 0.004	1.780 ± 0.055	57.22 ± 0.50	40.56 ± 0.50	2.21 ± 0.10
	3	0.378 ± 0.002	1.648 ± 0.019	68.20 ± 0.17	29.00 ± 0.17	2.79 ± 0.043
	4	0.449 ± 0.004	1.334 ± 0.012	41.33 ± 0.19	39.61 ± 0.20	18.3 ± 0.59
	4 + No. H *	0.008 ± 0.456	1.510 ± 0.013	40.01 ± 0.94	42.79 ± 0.86	17.2 ± 0.42
**7 g Si Powder + 4.5 wt.% KOH**	2	0.351 ± 0.004	1.78 ± 0.055	57.2 ± 0.50	40.6 ± 0.50	2.21 ± 0.10
	3	0.369 ± 0.006	1.73 ± 0.043	63.3 ± 0.93	34.5 ± 0.93	2.18 ± 0.10
	4	0.372 ± 0.002	2.19 ± 0.026	55.8 ± 0.53	41.7 ± 0.53	2.45 ± 0.03
	4 + No. F *	0.470 ± 0.009	1.54 ± 0.014	36.6 ± 0.83	44.2 ± 0.70	19.3 ± 0.43
**7 g Si Powder + 3 wt.% KOH**	2	0.376 ± 0.009	1.58 ± 0.080	61.0 ± 1.40	36.0 ± 1.5	2.79 ± 0.20
	3	0.358 ± 0.004	1.97 ± 0.038	58.9 ± 1.00	38.8 ± 1.0	2.32 ± 0.08
	4	0.373 ± 0.007	1.86 ± 0.050	64.4 ± 1.00	33.3 ± 1.0	2.33 ± 0.11
	4 + No. F *	0.497 ± 0.011	1.42 ± 0.015	43.9 ± 0.88	37.1 ± 0.8	18.9 ± 0.50
**5 g Si Powder + 3 wt.% KOH**	2	0.351 ± 0.003	2.15 ± 0.05	57.7 ± 0.95	40.06 ± 0.95	2.28 ± 0.07
	3	0.353 ± 0.004	1.63 ± 0.05	62.7 ± 0.48	35.66 ± 0.48	1.63 ± 0.07
	4	0.382 ± 0.015	2.26 ± 0.026	64.4 ± 0.42	32.59 ± 0.42	2.99 ± 0.04
	4 + No. F *	0.481 ± 0.014	1.31 ± 0.014	41.0 ± 0.89	37.49 ± 0.91	21.47 ± 0.6

* No. H: without heat treatment, No. F: without filtration.

## Data Availability

The data presented in this study are available on request from the corresponding author.
